# Growth rate control of flagellar assembly in *Escherichia coli* strain RP437

**DOI:** 10.1038/srep41189

**Published:** 2017-01-24

**Authors:** Martin Sim, Santosh Koirala, David Picton, Henrik Strahl, Paul A. Hoskisson, Christopher V. Rao, Colin S. Gillespie, Phillip D. Aldridge

**Affiliations:** 1 Centre for Bacterial Cell Biology, Baddiley Clark Building, Newcastle University, Richardson Road, Newcastle upon Tyne, NE2 4AX, United Kingdom; 2 Institute for Cell and Molecular Biosciences, Newcastle University, Framlington Place, Newcastle upon Tyne, NE2 4HH, United Kingdom; 3 Department of Chemical and Biomolecular Engineering, University of Illinois at Urbana-Champaign, Urbana, Illinois, 61801, United States; 4 Strathclyde Institute of Pharmacy and Biomedical Sciences, University of Strathclyde, Glasgow G4 0RE, United Kingdom; 5 School of Mathematics & Statistics, Herschel Building, Newcastle University, Newcastle upon Tyne, NE1 7RU, United Kingdom

## Abstract

The flagellum is a rotary motor that enables bacteria to swim in liquids and swarm over surfaces. Numerous global regulators control flagellar assembly in response to cellular and environmental factors. Previous studies have also shown that flagellar assembly is affected by the growth-rate of the cell. However, a systematic study has not yet been described under controlled growth conditions. Here, we investigated the effect of growth rate on flagellar assembly in *Escherichia coli* using steady-state chemostat cultures where we could precisely control the cell growth-rate. Our results demonstrate that flagellar abundance correlates with growth rate, where faster growing cells produce more flagella. They also demonstrate that this growth-rate dependent control occurs through the expression of the flagellar master regulator, FlhD_4_C_2_. Collectively, our results demonstrate that motility is intimately coupled to the growth-rate of the cell.

The flagellum is a rotary motor that enables bacteria to swim in liquids, swarm over surfaces and aid attachment to surfaces^1^. Some bacterial species employ a single flagellum for motility whereas others utilise multiple flagella. *Escherichia coli* is a prominent example of a bacterium that employs many flagella^2^. This bacterium produces 5–10 flagella that are randomly distributed across the cell surface. By altering the rotational direction of these flagella, *E. coli* is able to swim towards attractants and away from repellents via a biased random walk alternating between runs and tumbles^2^.

The flagellum is a complex organelle requiring the coordinated expression of over fifty genes^3^. Numerous regulators have been shown to control flagellar gene expression in *E. coli*^4^. Key among these is the *flhDC* operon, encoding the FlhD_4_C_2_ transcriptional regulator^5^,^6^. This regulator is essential for flagellar assembly, and it sits atop a transcriptional hierarchy that couples transcriptional activity to macromolecular assembly^3^. Global regulators are known to affect the expression and activity of FlhD_4_C_2_ in *E. coli*. These regulators are thought to coordinate flagellar gene expression with cellular and environmental factors such as nutrient availability, temperature, osmolarity, and envelope stress^4^. In addition, *flhDC* expression is known to vary during cell growth, where expression is greatest during mid-log phase^7^. These results suggest that motility is coupled to the growth-rate of the cell. However, a systematic study has not yet been described under controlled growth conditions.

In the present study, we investigated the extent to which the growth-rate of the cell controls flagellar formation in *E. coli* in steady-state chemostat cultures, where we can precisely control the growth-rate of the cell^8^. Our results demonstrate that growth-rate impacts flagellar abundance in that faster growing cells produce more flagella. Our data suggest that this growth-rate dependent control occurs via changes in the expression of the flagellar master regulator, FlhD_4_C_2_. We conclude that this intimate relationship between growth-rate and flagellar abundance indicates that cells exploit flagella for more than just foraging for food.

## Results

**Figure 1:**
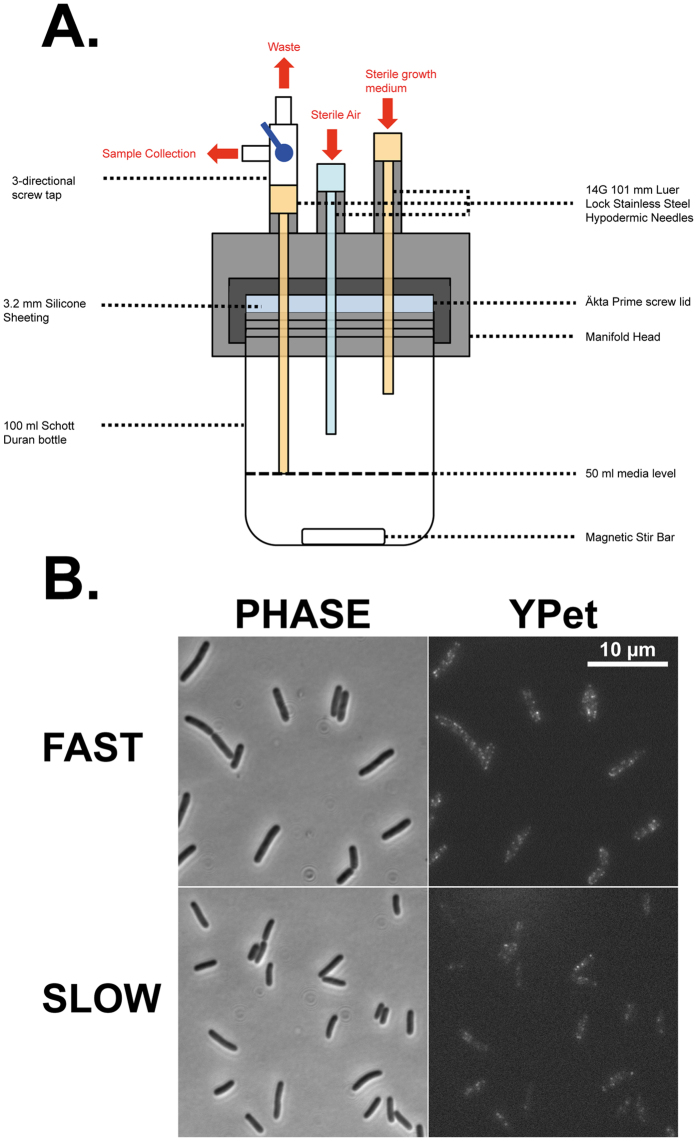
(**A**) Schematic diagram of the chemostat system developed for this study. (**B**) Example phase contrast and fluorescent images taken from wild-type in both the fast and slow conditions. All images are to scale with the scale bar showing 10 μm.

The specific growth rate in bacterial populations, μ, is expressed in reciprocal hours (hr^−1^) and is calculated, in batch cultures, from the slope of the semi-logarithmic phase of growth^9^ and in continuous, chemostat, culture is equal to the dilution rate^8^. Here we first measured the number of flagella in *E. coli* during fast (μ = 0.6 hr^−1^) and slow (μ = 0.12 hr^−1^) growth in steady-state chemostat cultures (Fig. 1A; see Materials and Methods for details). These growth-rates are equivalent to 1.2 hour and 5.8 hour doubling times, respectively^10^. To count the number of flagella in the cell, we utilized a strain expressing a functional fusion of FliM, which forms the inner C-ring of the flagellum, to the yellow fluorescent protein variant, YPet^11^. These fusions form distinct foci in the cells that can be used to visualize individual flagella (Fig. 1B). By counting the number of FliM-YPet foci in a single focal plane using fluorescence microscopy, we were able to estimate the number of flagella that individual cells express. Using microscopy allowed our analysis to incorporate a high number of individual cells for all conditions and mutants tested. Our analysis then allowed for the distribution of foci to be determined in an accumulative number of cells captured across 3 independent biological repeats of each experiment.

**Figure 2:**
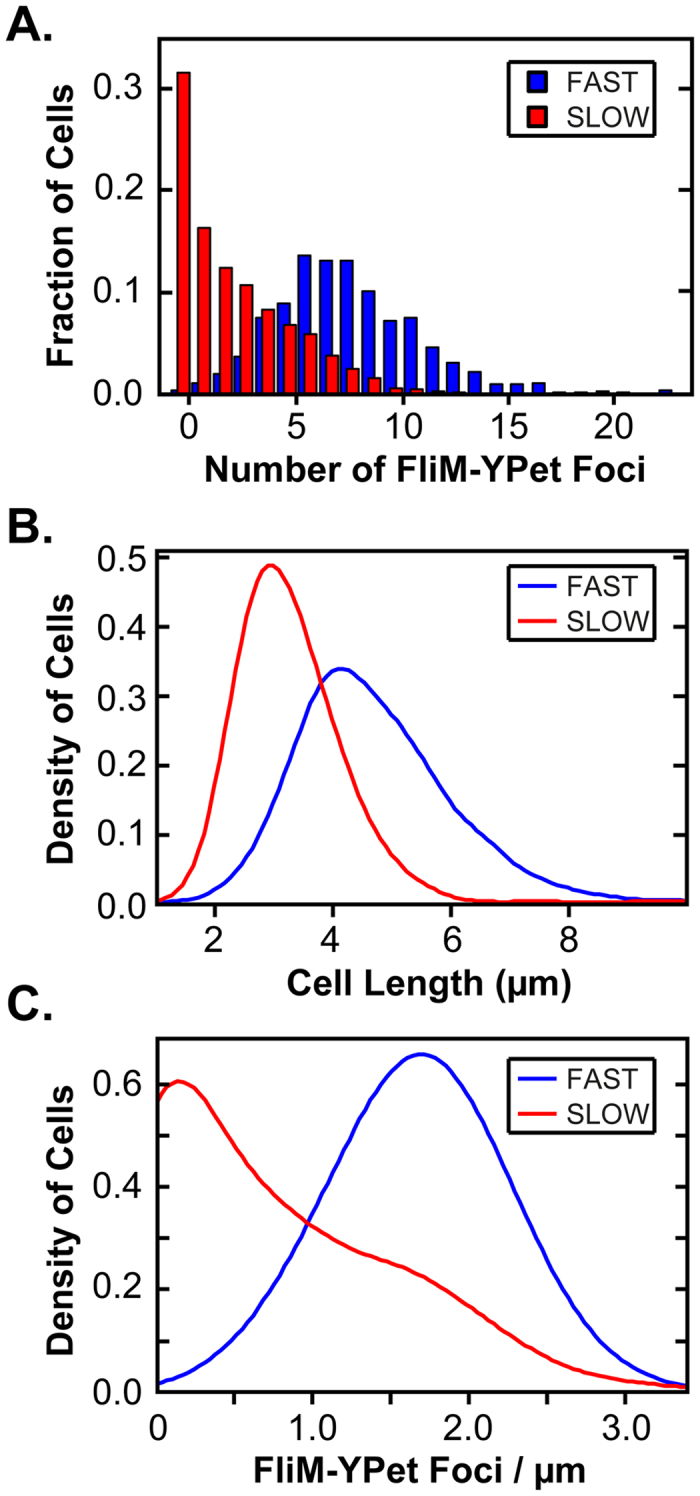
Growth rate affects flagellar assembly in the wild-type. (**A**) Number of foci in individual cells during fast (blue; n = 1099 cells) and slow (red; n = 1971 cells) growth. (**B**) Cell length during fast (blue) and slow (red) growth. (**C**) Number of foci/μm during fast (blue) and slow (red) growth.

Figure 2A shows the distribution of foci per cell during fast and slow growth. During fast growth, an approximately symmetrical distribution is observed with a mean of 7.8 foci per cell. During slow growth, an exponential-type distribution is observed with a mean of 2.4 foci per cell. These results demonstrate that the flagellar abundance is correlated with the growth rate of the cell, with faster growing cells on average expressing more flagella. One potential explanation is that these differences are due to cell length. Indeed, cells are much longer during fast growth (Fig. 2B), with a mean length of 4.7 μm during fast growth versus 3.3 μm during slow growth. To account for these differences in cell length, we also compared the number of foci per μm cell length (foci/μm) (Fig. 2C). Once again, we observed more foci/μm in fast growing cells (mean = 1.7 foci/μm) than in slow growing cells (mean = 0.7 foci/μm). In addition, the distribution shapes were significantly different, where the fast growing cells again exhibited a near symmetrical shape, suggesting a normal distribution, and the slow ones a sloped shaped distribution trending towards low flagellar abundance. Collectively, these results demonstrate the fast growing cells express more flagella than slow growing ones.

**Figure 3:**
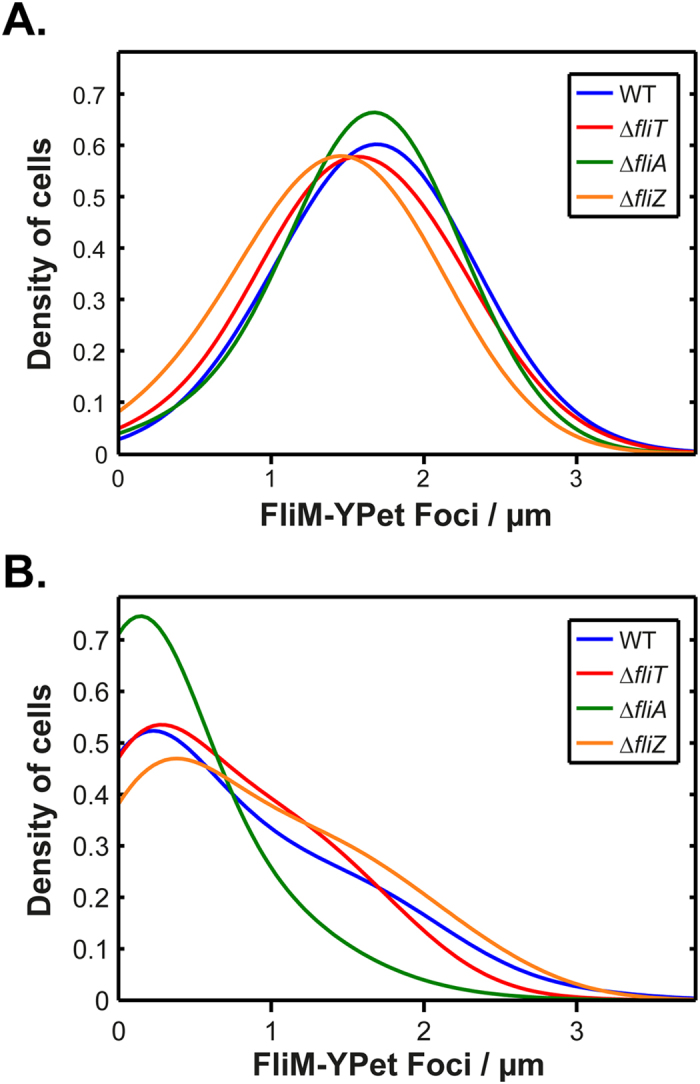
Flagellar regulators do not affect the growth-rate dependent control of flagellar assembly. (**A**) Foci/μm during fast growth in the wild-type (n = 1099 cells), ∆*fliT* (n = 368 cells), ∆*fliA* (n = 861 cells), and ∆*fliZ* (n = 877 cells) mutants. (**B**) Foci/μm during slow growth in the wild-type (n = 1971 cells), ∆*fliT* (n = 905 cells), ∆*fliA* (n = 1174 cells), and ∆*fliZ* (n = 1339 cells) mutants. The legends in (**A**) and (**B**) show the line colour representing each mutant.

We next explored the regulatory factors governing the growth-rate control of flagellar abundance. We first focused on the downstream flagellar-specific regulators known to affect abundance in enteric bacteria^10^,^12^,^13^,^14^. We chose to analyse the impact FliA, FliT and FliZ have upon our identified phenotype. FliA encodes the flagellar specific sigma factor σ^28^ and controls late gene expression^15^. FliT is known in *Salmonella* to disrupt the FlhD_4_C_2_ complex reducing its availability to interact with DNA^10^. In *E. coli* FliZ directly interacts with the *flhDC* 5′ untranslated region^16^. Figure 3 shows the distribution of foci/μm in the wild type as compared to ∆*fliT*, ∆*fliA*, and ∆*fliZ* deletion mutants during fast and slow growth. Under both conditions, the distributions are similar for the deletion mutants and the wild type. In general, we observed that all deletion mutants exhibited a similar growth-rate response with a minor reduction in the number of flagella in the deletion mutants compared to the wild type at the fast growth-rate. Two exceptions were observed under the slow conditions i) a small increase in the number of foci/μm in the ∆*fliZ* mutant compared to the wild type (0.9 versus 0.7 foci/μm) and ii) a significant decrease in the mean foci/μm in the ∆*fliA* mutant (0.3 versus 0.7 foci/μm). Evidence suggests that FliA positively regulates a number of *flhDC* dependent promoters by recruiting core RNA polymerase^17^. The phenotype of ∆*fliA* we observe is consistent with the observations of Liu and Matsumura 1996. However, the distribution shapes are similar with all deletion mutants responding to growth rate changes. These results demonstrate that growth-rate control is likely not due to the downstream flagellar specific regulators but rather subject to global regulation.

**Figure 4:**
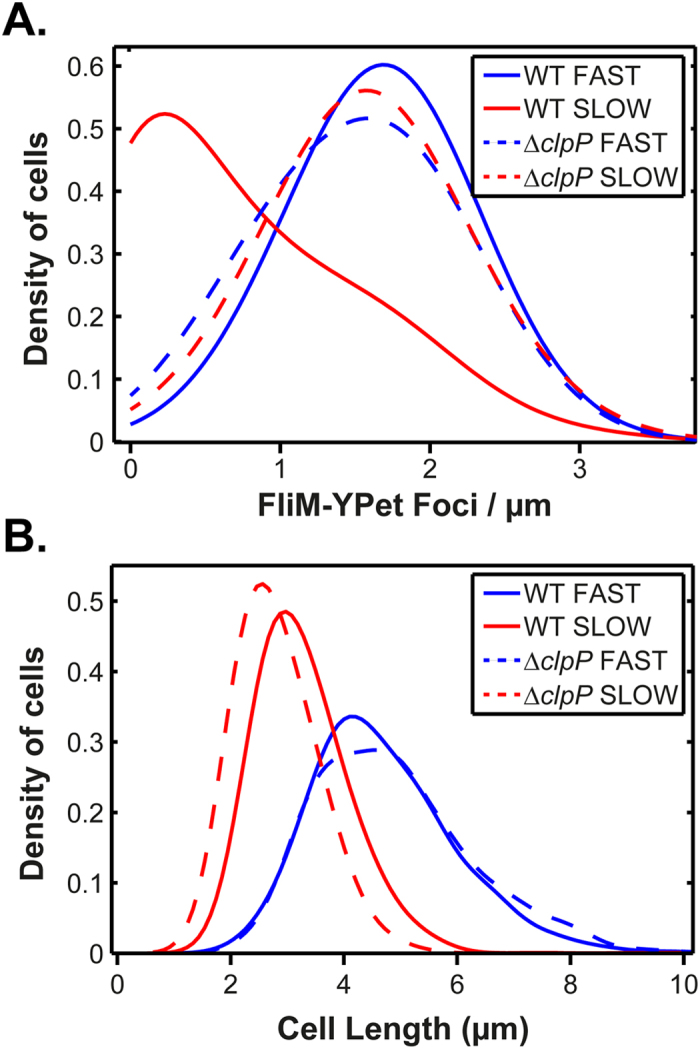
ClpP is necessary for growth-rate dependent control of flagellar assembly. (**A**) Foci/μm during fast and slow growth in the wild-type (n = 1099 (fast) and n = 1971 (slow) cells) and a ∆*clpP* mutant (n = 718 (fast) and 1365 (slow) cells). (**B**) Cell length during fast and slow growth in the wild-type and a ∆*clpP* mutant. Line styles and colours are explained in each legend.

A number of studies have shown that the ClpXP protease regulates flagellar assembly by degrading FlhD_4_C_2_^18^. We hypothesized that growth-rate control may be due to ClpXP. Consistent with this notion, we observed differences in the distribution of foci/μm during slow growth in a ∆*clpP* deletion mutant as compared to the wild type (Fig. 4A). Specifically, the shape of the distribution is no longer of an exponential-type but rather resembles a symmetrical distribution, similar to what is observed during fast growth. Indeed, when we compare the ∆*clpP* deletion mutant during fast and slow growth (Fig. 4A), the distributions are similar to each other and to the wild type during fast growth. We note that the wild-type cells still exhibit more foci/μm than the ∆*clpP* deletion mutant (mean = 1.7 versus 1.5) during fast growth. However, during slow growth, wild-type cells exhibit fewer foci/μm than the ∆*clpP* deletion mutant (mean = 0.7 versus 1.6).

Cells are shorter during slow growth as compared to fast growth (Fig. 2B). To test whether the ∆*clpP* results are due to altered cell length, we compared cell length in the wild-type and the ∆*clpP* deletion mutant during fast and slow growth. As shown in Fig. 4B, the cell length distributions for the ∆*clpP* deletion mutant do not significantly deviate from the wild-type. The only notable difference is that the ∆*clpP* deletion mutant are somewhat shorter than the wild type during slow growth (2.8 versus 3.3 μm). These results demonstrate that the ∆*clpP* effect is not caused by changes in cell length.

Our results suggest that ClpP is necessary for the growth-rate control of flagellar assembly. However, we cannot conclude from these data that ClpP is acting alone to regulate the flagellar system in response to growth rate. What we can conclude is that growth-rate control likely occurs through FlhD_4_C_2_ because: 1) down-stream flagellar regulators do not affect foci distributions, and 2) ClpXP is known to principally target FlhD_4_C_2_ within the flagellar regulon. Control through FlhD_4_C_2_ can occur either by regulating its production or degradation in a growth-rate dependent manner. To determine which mode is affected by the growth rate of the cell, we compared the foci distribution in the wild type and a strain where the native P_*flhDC*_ promoter was replaced with a tetracycline-inducible promoter (P_*flhDC*_::P_*tetRA*_). Using this strain, we can fix the level of *flhDC* expression, using derivatives of tetracycline at concentrations chosen so that induction matched the wild type during fast growth^12^,^14^,^19^.

**Figure 5:**
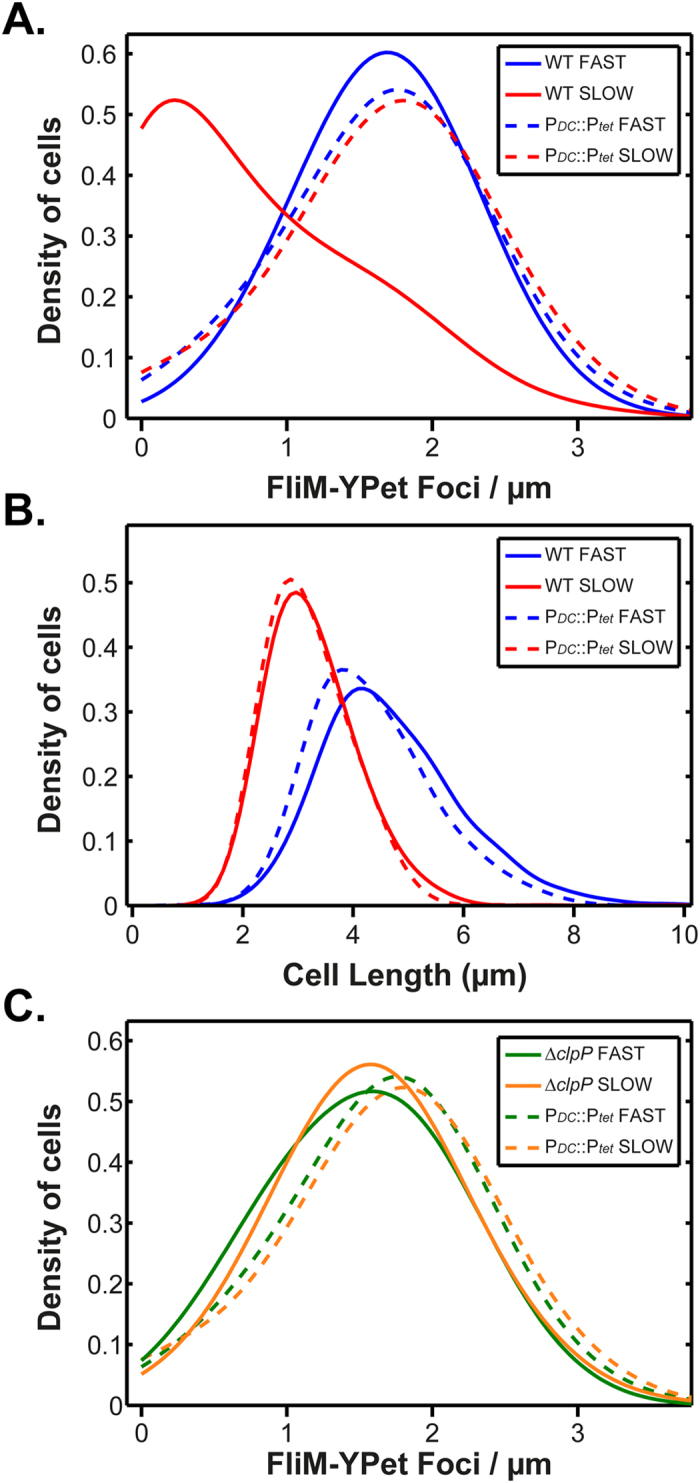
Growth-rate control of flagellar assembly occurs at the level of *flhDC* transcription. (**A**) Foci/μm during fast and slow growth in the wild-type (n = 1099 (fast) and n = 1971 (slow) cells) and a P_*flhDC*_::P_*tetRA*_ mutant (n = 743 (fast) and 1470 (slow) cells). (**B**) Cell length during fast and slow growth in the wild-type and a P_*flhDC*_::P_*tetRA*_ mutant. (**C**) Foci/μm during fast and slow growth in P_*flhDC*_::P_*tetRA*_and ∆*clpP* mutants. Line styles and colours are explained in each corresponding legend. P_*flhDC*_::P_*tetRA*_ is abbreviated to P_*DC*_::P_*tet*_ in the legends.

As shown in Fig. 5A, constitutive expression of *flhDC* removes the growth-rate dependence of flagellar assembly. The foci distributions during fast and slow growth in the P_*flhDC*_::P_*tetRA*_ strain are similar. In addition, both distributions are similar to the wild-type distributions during fast growth. As before, the effect is not due to cell length, because the cell length distributions of the P_*flhDC*_::P_*tetRA*_ strain match the wild type during both fast and slow growth (Fig. 5B). We also compared the foci distribution during fast and slow growth in the P_*flhDC*_::P_*tetRA*_ strain and ∆*clpP* deletion mutant. As shown in Fig. 5C, the distributions are nearly identical. Based on these results, we conclude that growth-rate control of flagellar assembly principally occurs at the level of *flhDC* transcription. Deleting *clpP* likely compensates for weak *flhDC* expression during slow growth by reducing its degradation rate, thereby increasing overall FlhD_4_C_2_ concentrations. Moreover, if growth rate control was solely due to ClpXP (we cannot discount that it does not contribute) then we would expect that the P_*flhDC*_::P_*tetRA*_ strain and wild type would have similar distributions during slow growth.

FliM is an integral component of the C-ring associating with the base of the flagellar MS-ring within the inner membrane^2^. Previous studies have hinted that the number of FliM foci in *E. coli, Salmonella and Bacillus* may not correlate to functional, fully assembled flagella^20^,^21^,^22^. The ability of maleimide to crosslink free cysteine has been frequently exploited to label the flagellar filament and flagellar hook^22^,^23^,^24^. To correlate FliM foci to functional flagella in our assay conditions and analysis, we introduced a hook specific *flgEA240C* amino acid exchange into the chromosome of strain JPA945 used in this study (see supporting material for details).

**Figure 6:**
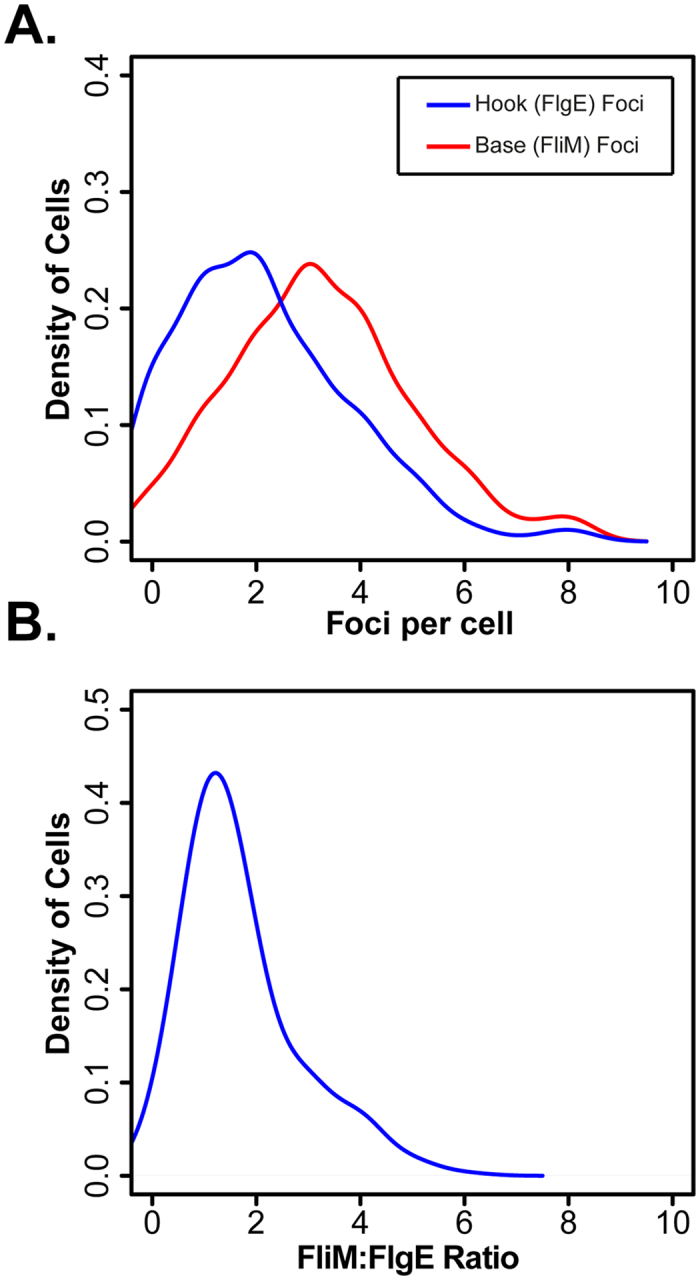
Base (FliM) and hook (FlgE) foci correlate while showing that not every base maybe a functional flagellum. (**A**) Distribution of base and hook foci in batch culture growth conditions. (**B**) Distribution of the base:hook ratio taken from the individual cells used in (**A**). The data shown is n = 328 cells from 3 independent repeats of experiments where cells were grown in media containing 3 g/L yeast extract generating an equivalent growth rate during exponential growth as used for fast growth conditions in the chemostat experiments^10^. Images and further details of the generation of *flgEA240C* can be found in the supporting material.

Using batch culture, rather than chemostat conditions, we investigated the ratio of flagellar bases to functional hooks in fast growth conditions. Analysis of the distribution of bases and hooks shows that the hook distribution has a mean value lower than that observed for bases (Fig. 6A). Importantly, the majority of cells analysed have a base:hook ratio of 1:1 or 2:1 with a bias towards 1:1 in our assay conditions (Fig. 6B).

## Discussion

Multiple studies have shown that diverse cellular parameters are correlated with the growth rate of the cell. In the present study, we demonstrated that flagellar abundance is correlated with growth rate, with faster growing cells producing more flagella than slower growing ones. These results are not due to changes in cell length: the same trends hold when we normalize flagellar abundance by cell length. By characterizing different mutants, we were further able to show that the growth-rate control of motility occurs principally through FlhD_4_C_2_. In particular, replacing the native P_*flhDC*_ promoter with a constitutive one removes the growth rate control of flagellar assembly. These results demonstrate that *flhDC* expression is responsive to the rate of cell growth, and thus provide a key mechanism for the growth-rate control of flagellar abundance.

Key to our analysis was the use of chemostats for the precise control of the steady-state growth rate of the cell. The use of chemostats allows for the decoupling of growth-rate from secondary physiological effects such as nutrient limitation and stress^8^. While previous studies have also investigated the growth-rate control of motility, they were performed in batch cultures where the growth-rate is not stable but instead varies along the growth curve^7^. Another distinct aspect of our work was that we measured flagellar abundance in individual cells using fluorescence microscopy^10^,^11^,^25^. This allowed for analysis of a large population of cells across independent biological repeats. We further show that the base:hook ratio defined by FliM and FlgE foci are in agreement. To do this we exploited the crosslinking properties of maleimide allowing us to use a similar assay and image capture conditions as used for the chemostat experiments.

Importantly our data show that flagellar production is a stochastic process with significant variability among individual cells. Moreover, the shapes of the distributions change significantly during fast and slow growths in our chemostat-based experiments. These shapes are also consistent with slow growing cells producing flagella more infrequently, due to weaker FlhD_4_C_2_ expression, than fast growing ones^26^.

Flagellar biosynthesis is known to be subject to glucose catabolite repression in *E. coli* through the action of CRP^27^. The general explanation is that cells are motile only when nutrients are limiting, as expected if motility were employed solely for foraging^28^. Our results offer a more complex mechanism by showing that flagellar biosynthesis is also linked to the growth-rate of the cell. In particular, nutrients are more limiting during slow growth than fast growth, and others have shown that cyclic AMP concentrations are inversely correlated with the growth-rate^29^. If catabolite repression were the dominant mechanism, then one would expect more flagella during slow growth, contrary to what we observe. This suggests that there is likely a layer of regulation that supersedes catabolite repression and is masked somehow during batch growth (or, alternatively, catabolite repression is masked somehow during chemostat growth). The identity of these regulators and associated signals is not currently known.

We note only a single strain of *E. coli* was investigated in the present study. Whether the same results hold in other strains is unknown. A previous study demonstrated that many K-12 laboratory *E. coli* strains contain insertion sequence (IS) elements upstream of the *flhDC* operon and that strains lacking these elements are poorly motile. *E. coli* RP437, the strain investigated in this study, contains an IS5 element upstream of the P_*flhDC*_ promoter^30^. Other strains, such as MG1655 (seq), contain an IS1 element^30^. To what degree these elements contribute to the growth-rate control of flagellar biosynthesis is unknown.

What possible advantages accrue from the growth-rate control of motility? The simplest explanation is that slow growing cells lack the resources to produce numerous flagella and must settle instead for producing just a few. Another related argument is that cells need to balance flagellar production with growth so that their progeny have sufficient flagella^31^. In other words, if fast growing cells are not producing flagella with a high enough rate, then some daughter cells may lack flagella. An alternative explanation is that growth-rate control integrates the crosstalk recognised to coordinate the response of other macromolecular systems such as pili and efflux in enteric systems^32^,^33^. However, the observed increase in flagella production is greater than would be necessary for balancing production and growth: when we normalize the number of flagella by the length of the cell, fast growing cells are still producing flagella at a greater rate. This would argue in favour of the first explanation that slow growing cells must settle on producing fewer flagella. Our data therefore suggests that motility is employed for reasons other than just foraging as previously proposed^1^,^2^,^3^,^4^.

## Materials and Methods

### Bacterial strains and growth conditions

Strains used or constructed in this study are shown in Table 1. Overnight pre-cultures to inoculate chemostats were grown in LB media at 37 °C with constant shaking at 180 rpm. Antibiotics were used as previously described^34^. Autoclaved chlortetracycline was used to induce *flhDC* expression at 2.5 μg/ml^35^. Bacterial strains were created according to the lambda-red recombination gene knockout strategy of Datsenko and Wanner^36^. Antibiotic resistance cassettes were amplified by PCR from plasmids pKD3 or pKD4. Oligonucleotide sequences used to generate deletion mutants and *flgEA240C* are available on request. Lambda-red expression was induced with 0.1% arabinose when cultures reached an OD_600_ = 0.1. Cells were prepared for electroporation once the induced cultures had reached an OD_600_ = 0.6–0.8 at 30 °C. Colonies were checked for insertion of the appropriate resistance cassette through colony PCR and phenotypic analysis using motility agar. P_*flhDC*_::P_*tetRA*_ mutants were phenotypically screened for tetracycline resistance and motility assays after 8 hrs incubation at 30 °C with and without tetracycline in the motility agar.

### Generation of *flgEA240C*

The details for identification of *flgEA240C* can be found in the supporting material. In brief putative cysteine codon switches were based on the location of *flgE2T242C* described by Schuhmacher *et al*.^23^. Two-step PCR was used to introduce the necessary mutations into the *flgE* coding sequence. On identification and confirmation, the pCRISPR-Cas system of Jiang *et al*.^37^ obtained from Addgene (plasmids #62226 and #62225) was utilised to introduce *flgEA240C* on to the chromosome. Instead of using a pTARGET-F derivative guide RNA directed to *flgE* we used an alternative strategy that allowed the isolation of functional insertions using motility agar as described previously^38^. The method required a *flgE* replacement using the *cat* gene from pKD3 then a *cat* specific guide RNA to drive CRISPR-Cas directed recombination. *flgEA240C* positive colonies were confirmed phenotypically using maleimide staining and sequencing.

### Chemostat continuous culture

Chemostat equipment was assembled and performed within a temperature controlled warm room at 30 °C. Media was comprised of Minimal E-salt medium^39^ supplemented with 1 g L^−1^ yeast extract and 0.2% glucose. The chemostat equipment consisted of 100 ml Duran Bottles containing a single 20 × 6 mm magnetic stirrer sealed with a GE Healthcare Akta Prime screw top lid and 3.2 mm silicone (Silex). Hypodermic needles (14 g × 4 inches) were inserted and held in place using a custom made attachment over the bottle lid (Fig. 1A). Hypodermic needles were connected with tubing to a sterile air supply provided by an aquarium pump, a three-way tap for sample collection and waste efflux and to a fresh media supply via a peristaltic pump (VWR). Culture vessels had a working volume of 50 ml and were inoculated with bacterial cultures to a starting OD_600_ of 0.05. Cultures were grown under batch conditions for 3 hours until reaching an OD_600_ of ~0.6. The peristaltic feeding pump was then switched on. Dilution rates were 0.5 ml/min for ‘fast’ growth and 0.1 ml/min for ‘slow’ growth. Steady-state growth was obtained after five volumes of media had passed through the vessel at each defined dilution rate. Samples were collected after steady-state had been reached, the OD600 recorded and the samples assessed by Fluorescent microscopy. Experiments were performed as equilibration to the fast growth rate then re-equilibrated to the slow growth rate. All experiments were performed at least 3 times.

### Fluorescence microscopy

Microscopy was performed using 1% agarose pads on multispot microscope slides (Hendley-Essex). Bacterial cultures were diluted to an OD_600_ of 0.5–0.8 to allow sufficient separation of cells in the field of view and one microliter spotted and allowed to air dry on the agarose surface. Microscopy was performed using a Nikon Ti inverted microscope using a Sutter Instruments Lambda LS light source and a Nikon 100 × 1.30 oil objective coupled to a Photometrics CoolSNAP HQ CCD camera. The system was controlled and images acquired with MetaMorph v7.7.80 software, aided by Nikon Perfect Focus system. Phase contrast images were obtained using an exposure time of 100 ms and YPet at 1000 ms. Staining using AlexaFluor568-maleimide was performed as previously described using a 1000 ms exposure^23^.

### Image Analysis using MicrobeTracker

Microscopy images captured were processed using MicrobeTracker^40^ within the current builds of MatLab (Mathworks). Cells detected on phase contrast channel images using the supplied alg4ecoli.set parameter. Cells were confirmed for accurate detection and manually split or joined as appropriate. The SpotFinderZ application within MicrobeTracker was utilised to detect fluorescent foci on the fluorescent image channel within cell co-ordinates saved from the phase contrast channel images. MicrobeTracker measures cell length in pixels automatically. Cell length was converted from pixels to micrometers using a conversion factor calculated with a CS1358 micrometer (Graticules Ltd). The resulting data analysis used custom Matlab scripts. The probability distributions in Figs 2, 3, 4, 5 and 6 were estimated using kernel density estimation with a Gaussian kernel in either Matlab or R.

## Additional Information

**How to cite this article:** Sim, M. *et al*. Growth rate control of flagellar assembly in *Escherichia coli* strain RP437. *Sci. Rep.* **7**, 41189; doi: 10.1038/srep41189 (2017).

**Publisher's note:** Springer Nature remains neutral with regard to jurisdictional claims in published maps and institutional affiliations.

## Supplementary Material

Supporting Material

## Figures and Tables

**Table 1:** *Escherichia coli* strains used or created during this study.

Strain number	Genotype	Source
JPA 945	*fliM-ypet* in RP437	11
TPA 3612	*fliM-ypet* ∆*clpP::*FRT-*cat*-FRT	This study
TPA 3613	*fliM-ypet P*_*flhDC*_*::P*_*tetRA*_	This study
TPA 3648	*fliM-ypet ∆fliT::*FRT-*cat*-FRT	This study
TPA 3653	*fliM-ypet ∆fliA::*FRT-*npt*-FRT	This study
TPA 3676	*fliM-ype*t *∆fliZ::*FRT-*npt*-FRT	This study
TPA 4675	*fliM-ype*t *flgEA240C*	This study
