# Supplementary material for: Growth rate control of flagellar assembly in Escherichia coli strain RP437
Source: Sci Rep. 2017 Jan 24;7:41189. doi: 10.1038/srep41189 (PMC5259725; doi:10.1038/srep41189)
Supplement: Supporting Material [file srep41189-s1.pdf]

Growth rate control of flagellar assembly in *Escherichia coli* strain RP437

Martin Sim[1,2], Santosh Koirala[4], David Picton[1,2], Henrik Strahl[1,2], Paul A. Hoskisson[5], Christopher V. Rao[4], Colin S. Gillespie[3], and Phillip D. Aldridge[1,2] *

Supplementary Information

Isolation of FlgEA240C

1: Centre for Bacterial Cell Biology, Baddiley Clark Building, Newcastle University, Richardson Road, Newcastle upon Tyne, United Kingdom, NE2 4AX.
2: Institute for Cell and Molecular Biosciences, Newcastle University, Framlington Place, Newcastle upon Tyne, United Kingdom, NE2 4HH
3: School of Mathematics & Statistics, Herschel Building, Newcastle University, Newcastle upon Tyne, United Kingdom, NE1 7RU
4: Department of Chemical and Biomolecular Engineering, University of Illinois at Urbana-Champaign, Urbana, Illinois, United States, 61801
5: Strathclyde Institute of Pharmacy and Biomedical Sciences, University of Strathclyde, Glasgow, United Kingdom G4 0RE

*Corresponding author. Centre for Bacterial Cell Biology, Baddiley Clark Building, Newcastle University, Richardson Road, Newcastle upon Tyne, United Kingdom, NE2 4AX. Phone: +44-191-2083218. Email: phillip.aldridge@ncl.ac.uk.

Supplementary Information – Isolation of FlgEA240C

Using maleimide conjugated fluorophores is becoming a common technique amongst the flagellar field [1-3]. One reason is that the majority of extracellular flagellar subunits do not contain cysteine residues.

Previous studies on *E. coli* have suggested that the FliM foci detected using FliM-Ypet are not all static. One argument for using FliM foci as a proxy for flagellar structures is that it avoids the technical issues of passing motile cells through a staining protocol with multiple steps that could potentially damage the external flagellar structure via shearing forces during centrifugation and pipetting. Even with these technical short comings of using staining techniques we have asked what is the ratio of FliM foci to FlgE foci?

Screening using a plasmid based copy of *flgE* amino acid substitutions A240C, A242C and T247C (**Figure S1**) generated in the same helix as the T242C change described previously[2] showed that A240C generated *flgE* foci (**Figure S2**).

The *flgEA240C* mutation was introduced in to *flgE* at its natural locus using CRISPR-Cas technology. To generate the *flgEA240C* substitution we exploited the pCas/Target-F system of Jiang et al (2015)[4] where pTarget-F was modified to target the *cat* gene of the Cm cassette from pKD3[5].

Isolation of *flgEA240C* was achieved using the isolation of motile recombinants using the method of Kakkanat *et al* (2015)[6] where 10 µl of a transformation was spotted on to motility agar.

RP437 *fliM-ypet flgEA240C* was grown to an OD600 of approx. 0.6 and single focal plane images were taken using the necessary filters to capture cells (Phase) FliM foci (YFP) and FlgE foci (RFP settings) (**Figure S3**). Images were then processed using Microbtracker using the same analysis as done for all chemostat experiments (**Figure 6**).

When images of FliM foci and FlgE foci are merged it is clear that using a single focal plane protocol identified foci do not always align to a base or a hook (**Figure S3** merged images). A comparison of *flgE*[+] and *flgEA240C* data with respect to the distribution of flagellar bases, defined by FliM foci, shows no impact of the A240C mutation on the distribution of FliM (**Figure S4**).

```
flgE2    220         ------------------------------TTLTFDAKGVLDPTTTKQAFDLTLPNGAS-
flgE1    239         GAAGTYQTDTDGDGVPDSTGTAETAGGWRGSVLKFNSVGVYTGSDPAIVTTETLGVGGAG
ecoli    223         KT---------------------------ATTLEFNANGTLVDGAMANNIATGAINGAE-
```

Figure S1 Alignment of FlgE from *E. coli* with *Shewanella* FlgE1 and FlgE2 indicating the T242C changed used by Schuhmacher et al (2015) compared to the residues substituted in FlgE from *E. coli* in this study.

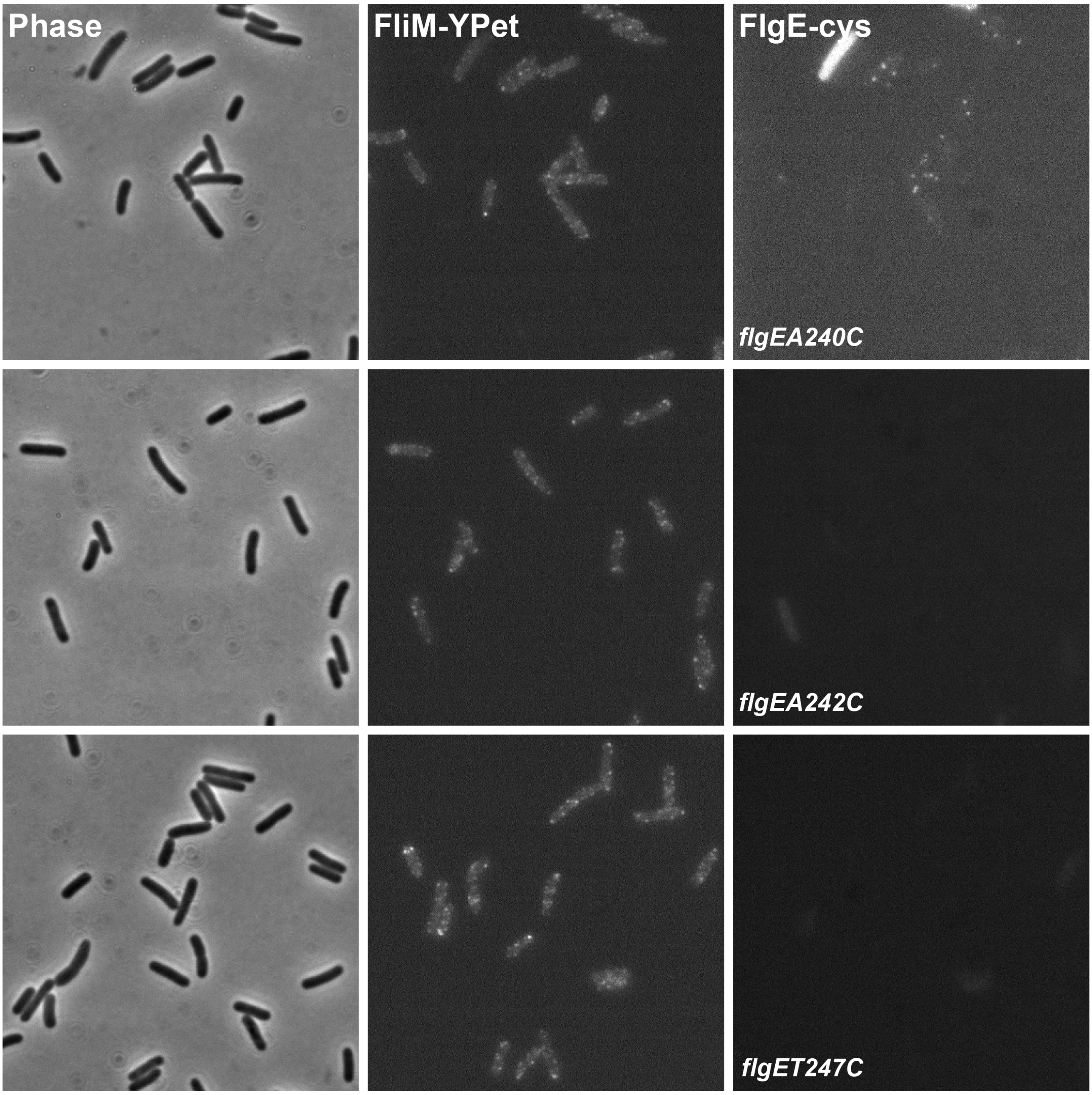

Figure S2 Screening *flgE* cys substitutions expressed from pBAD30 with arabinose induction in a Δ*flgE fliM*-ypet[+] deletion mutant using maleimide staining. Clear foci were only obtained with *flgEA240C* while A242C and T247C did not generate any visible foci. In contrast imaging of FliM-Ypet showed clear flagellar foci.

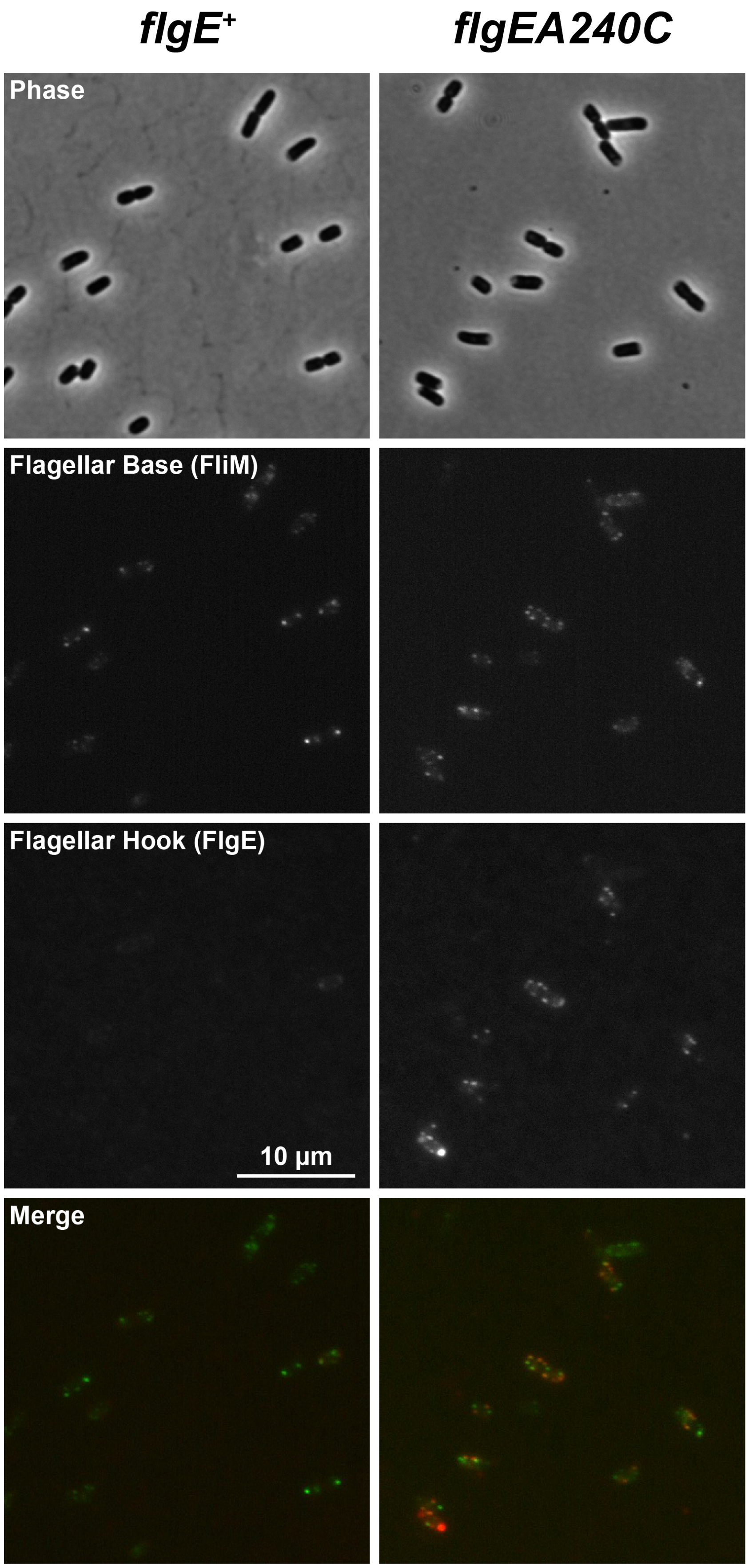

Figure S3: Summary of visualisation of the flagellar base and hook using FliM and FlgE foci in RP437 *fliM*-Ypet *flgEA240C* and RP437 *fliM*-Ypet *flgE*$^{+}$. Images are representative areas of fields of views used to generate the data in Figure 6 and Figure S4.

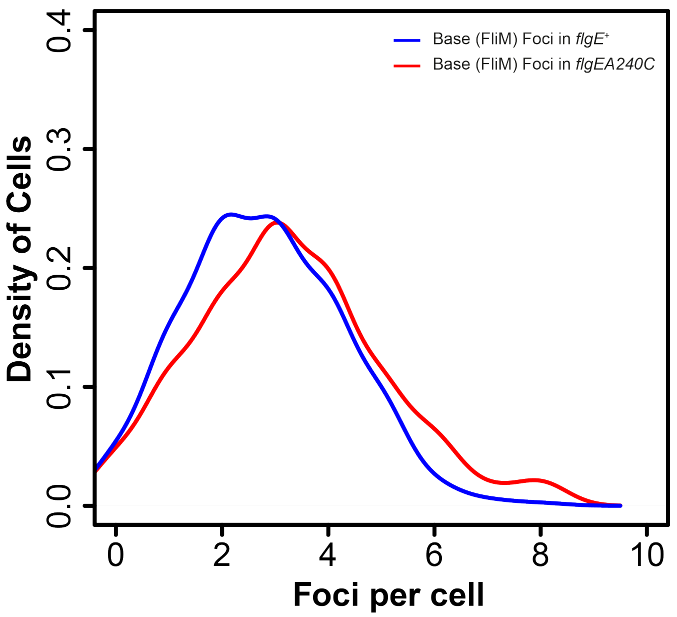

Figure S4: Comparison of FliM foci under the assayed growth conditions for *flgEA240C* and *flgE*$^{+}$ strains. This data shows that there is no impact of a *flgEA240C* allele on FliM foci.

1. Guttenplan, S. B., Shaw, S. & Kearns, D. B. The cell biology of peritrichous flagella in *Bacillus subtilis. Mol Microbiol* **87,** 211–229 (2012).
2. Schuhmacher, J. S. *et al.* MinD-like ATPase FlhG effects location and number of bacterial flagella during C-ring assembly. *Proc Natl Acad Sci USA* **112,** 3092–3097 (2015).
3. Turner, L., Zhang, R., Darnton, N. C. & Berg, H. C. Visualization of Flagella during bacterial Swarming. *J Bacteriol* **192,** 3259–3267 (2010).
4. Jiang, Y. *et al.* Multigene Editing in the *Escherichia coli* Genome via the CRISPR-Cas9 System. *Appl Environ Microbiol* **81,** 2506–2514 (2015).
5. Datsenko, K. A. & Wanner, B. L. One-step inactivation of chromosomal genes in *Escherichia coli* K-12 using PCR products. *Proc Natl Acad Sci USA* **97,** 6640–6645 (2000).
6. Kakkanat, A. *et al.* The role of H4 flagella in *Escherichia coli* ST131 virulence. *Sci Rep* 1–14 (2015).
